# The development of a Cannabis Knowledge Assessment Tool (CKAT)

**DOI:** 10.1371/journal.pone.0291113

**Published:** 2023-09-01

**Authors:** Ava Bayat, Holly Mansell, Jeff Taylor, Michael Szafron, Kerry Mansell

**Affiliations:** 1 College of Pharmacy and Nutrition, University of Saskatchewan, Saskatoon, SK, Canada; 2 School of Public Health, University of Saskatchewan, Saskatoon, SK, Canada; University of Alabama at Birmingham, UNITED STATES

## Abstract

**Background:**

Misconceptions about the health risks of cannabis remain prevalent, indicating the need to improve public health messaging and determine the effectiveness of educational programming. Our objective was to develop a standardized questionnaire to measure knowledge about cannabis in the context of cannabis legalization.

**Methods:**

A Cannabis Knowledge Assessment Tool (CKAT) was created using the Delphi method. A purposive sample of healthcare professionals, policymakers, academics, patients, and students served as the content and development experts. Principal component analysis from the codes identified from open-ended feedback guided the item development. Upon completion, the CKAT was administered as a pre- and post-test in four schools (7^th^ and 9^th^ Grade) in Canada. The data were analysed to determine whether knowledge scores changed after participating in a cannabis education program.

**Results:**

Twenty-four experts initially participated in the Delphi process and 18 (75% retention) continued throughout. Principal component analysis identified 3 domains: 1) effects of cannabis on the individual, 2) general information about cannabis, and 3) cannabis harm reduction. The final questionnaire consisted of 16 multiple-true-false questions (64 items) and received a Flesch-Kincaid Grade Level of 6.3, and a SMOG index score of 7.6. The CKAT was completed by 132 students; seventy-three 7^th^ grade and fifty-nine 9^th^ grade students. The baseline mean CKAT score was 46.2 (SD:5.5), which increased to 50.7 (SD:4.6) after the cannabis educational program (p<0.05).

**Conclusions:**

A novel tool to measure knowledge of cannabis was developed and piloted in 7^th^ grade and 9^th^ grade students. Future studies are required to test usability and validity of the CKAT in other contexts.

## Introduction

In 2018, Canada became the second country to introduce federal legislation providing a framework to allow adults to use cannabis recreationally [1]. The objectives of the *Cannabis Act*, which controls the federal production, distribution, and possession of cannabis, are to prevent young people from accessing cannabis, and protect public health and safety by establishing product requirements [2]. Other goals include deterring criminal activity (by imposing penalties for those operating outside the framework) and reducing the burden on the criminal justice system [2]. Opponents of cannabis legalization have raised various societal concerns, including the possibility of increased cannabis use in vulnerable populations (e.g., pregnant women, individuals with low socioeconomic status, or mental illness), and increased public harms such as cannabis-induced psychosis, and driving under the influence [3, 4].

After five years post-legalization, research on the impact of recreational cannabis in Canada is in its infancy. Some fears have not materialized (such as an increase in the prevalence of cannabis-induced psychosis) [5], whereas other indicators are more concerning. An increase in the prevalence of injured drivers testing positive for tetrahydrocannabinol (THC) has been noted post-legalization compared to pre-legalization [6]. Increases in cannabis-related visits to emergency departments, calls to poison control centers and unintentional pediatric cannabis ingestions have also been reported [7–10]. Misconceptions about the health risks of cannabis remain prevalent in the public, indicating the need to improve educational messaging [11]. Education is of particular importance for children, youth, and young adults due to their increased susceptibility for experiencing cognitive, social, and psychological deficits with regular cannabis use [12, 13]. Moreover, compared to other developed countries, Canadian youth report amongst the highest rates of cannabis consumption worldwide [14].

In the context of cannabis legalization, public awareness strategies are essential to support informed decision-making. People need to understand the benefits and risks of cannabis use, appropriate harm reduction strategies, and the laws and framework which govern the new legislation. However, there is a gap in the implementation of cannabis education across Canada [12], and the quantity and quality of drug education provided to youth remains inequitable [12, 15, 16]. Information taught about cannabis in schools has traditionally promoted abstinence, but the harms-based approach may no longer resonate with youth [12, 17]. Community-based programs that focus on harm reduction strategies have emerged to help fill this void [18, 19] however widespread adoption has not been implemented and the evaluation of such strategies has been limited.

The purpose of this study was to develop a standardized questionnaire to measure public knowledge about cannabis in the context of cannabis legalization, using the Delphi method. Such a tool could help identify misunderstandings and tailor educational resources to support specific populations. Since an objective measure of knowledge would also be useful for demonstrating the effectiveness of educational programs, we piloted the questionnaire in this context using a pre- and post-design.

## Methods

The project occurred over two phases (Questionnaire development and Questionnaire testing) and was approved by the Behavioural Regional Ethics Board at the University of Saskatchewan (BEH-1303 and BEH-1670, respectively).

### Phase 1: Questionnaire development

A Delphi method was utilized by way of an online survey platform (SurveyMonkey^®^) to develop the questionnaire (July 2019 to November 2019). This process builds consensus amongst a group of experts on a topic of interest using subsequent ‘rounds’ of questioning, whereby experts iteratively contribute feedback [20]. The process is completed once consensus is reached and no novel ideas are generated by participants. The initial questionnaire was created by the research team, which consisted of a Master of Science student and four faculty members and was piloted on three external graduate students. The research team collectively held expertise in the areas of cannabis education, knowledge assessment and questionnaire development and biostatistics.

#### Sampling and selection of Delphi experts

A purposive sample of experts (participants) consisting of healthcare professionals, policymakers, academics, people who take cannabis medically, and teenage students were recruited to aid in the development of the questionnaire. These groups were chosen for their unique perspective on what they feel is important for the public to know about cannabis. Healthcare professionals initially invited included pharmacists, nurses, harm reduction coordinators, and a physician with an interest in cannabis research or education, while policymakers consisted of law enforcement personnel, those employed by licensed producers, and advocates from relevant organizations that had a close familiarity with the novel rules and regulations surrounding cannabis use. Academics included professors with a focus on cannabis research and/or educational development. People who take cannabis medically provided perspective on their lived experience and students were included to ensure that the final questionnaire was relevant and understandable to potential test takers as low as the 7^th^ grade. An email was sent to prospective participants which explained the study and contained an embedded survey link for those who chose to participate. Completion of the surveys by participants implied free and informed consent. The identities of the participants were known to the research student and primary supervisor in case clarification was required or if a participant wished to withdraw any of their responses from the survey. No honorarium or incentive was provided.

#### Delphi process

**Fig 1:**
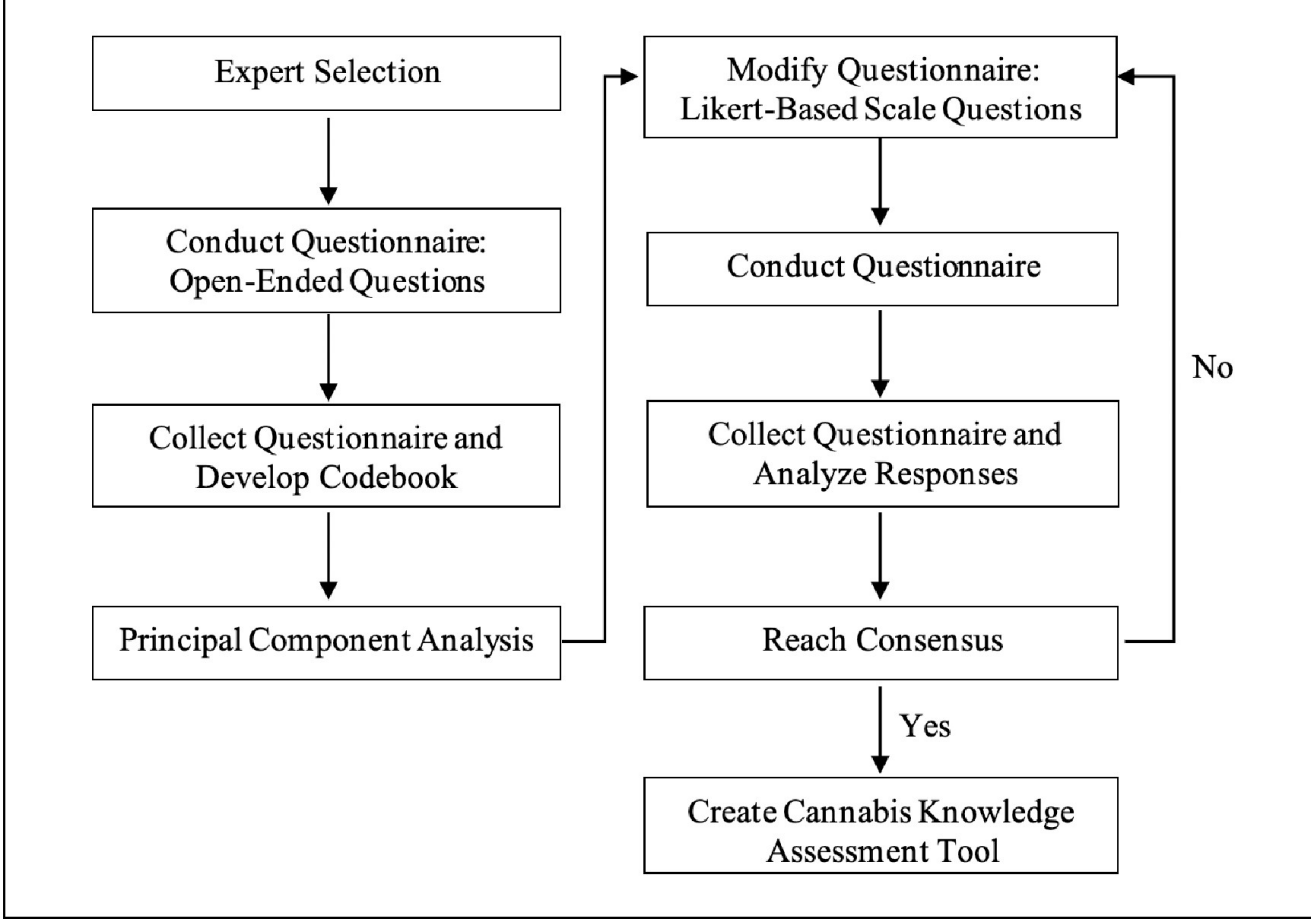
Delphi method process for development of questionnaire.

During each round of the survey, experts were emailed a link to a survey and were given a maximum of 4 weeks to provide feedback. A reminder was sent between 1 and 2 weeks after the initial email, and again at 3 weeks. This electronic format allowed for participation from a wide geographic region, and experts could respond freely and honestly; any potential power differential was eliminated since the participants were unbeknownst to each other. During each round, the data were collected, analyzed by the research team, and presented back to the experts in the next round for further feedback. All of the responses were amalgamated, and the data were presented anonymously with each round of the Delphi process. Consensus building emerged as further refinements were made to the questionnaire over the course of 3 rounds. The process is depicted in Fig 1.

#### Round 1

The first round of the Delphi process began by eliciting feedback from participants via the use of open-ended questions [21]. The experts were asked to identify topics the public should be expected to know about cannabis, common gaps in knowledge and misconceptions about cannabis, the appropriateness of questions for youth, and how much time it should ideally take to complete the questionnaire. Feedback from round 1 was initially analyzed qualitatively by AB and verified by HM and KM. Emergent coding, a process in which the categories emerge from the data itself rather than being pre-defined [22], was used to determine broad categories within the responses. Categories were subsequently dismantled into mutually exclusive meaning units or *codes* and recorded into a codebook developed in a Microsoft Excel spreadsheet. Principal component analysis (PCA) was subsequently performed using the codes generated from the question, ‘*What essential information should the general public know about cannabis*?*’* in IBM SPSS (version 26). PCA is a dimensionality reduction technique that allows researchers to identify a new component that is a combination of the old, larger set of variables [23]. As a result, a large data set (such as our codes, which were treated as variables in the analysis) could be transformed into a smaller set of variables (principal components). An initial draft of the questionnaire was created by the research team guided by the data identified in round 1. The questionnaire was presented in various formats (true and false, multiple choice, multiple true false) to two students in the 7^th^ grade and one student in the 9^th^ grade to elicit feedback on the type of format that would be best suited for the questionnaire.

#### Rounds 2–4

Rounds two through four of the Delphi process took the form of a structured questionnaire [21] whereby the experts were asked to review the list of proposed questions and state their agreement on whether each question should be included in the final questionnaire using a Likert-scale (*strongly agree*, *agree*, *neutral*, *disagree* or *strongly disagree)*. A threshold of 70% agreement (either *strongly agree* or *agree*) was used, whereby if less than 70% of the participants agreed the item was appropriate, it was removed or modified. Respondents were encouraged to supply feedback and suggestions for the improvement of each item. The revised questions were presented back to the group during each subsequent round in the Delphi method until final consensus was reached. The questionnaire was assessed for readability and revised to attain an 8^th^ grade reading level as per the Flesch-Kincaid formula and SMOG index [24, 25] prior to the final round of the Delphi method.

### Phase 2: Questionnaire testing

#### Study setting and questionnaire administration

Phase 2 of the study involved piloting the questionnaire to a cohort of students before and after their participation in a cannabis education program. A purposive sample of seven schools that had recently implemented the Real Education About Cannabis and Health (REACH) program [18] were used to test the responsiveness of the questionnaire. Grades 7 and 9 were chosen as the target age group because they represented time points in elementary and high school when students are likely to be introduced to cannabis, and these age groups were the targets of the REACH program. REACH is an educational program that was developed to provide students with evidence-based tools and knowledge required to understand and make healthy, informed decisions about cannabis use [18]. The REACH program consists of two modules that are consistent with the 7^th^ and 9^th^ grade Saskatchewan Ministry of Education’s health education curricular outcomes, respectively. Each module is comprised of four lessons: 1) Introduction to Cannabis, 2) The Science of Cannabis, 3) Social Science Implications and 4) Peer Pressure, Decision Making and Harm Reduction.

Prior to beginning the REACH program in 2020, the research team provided a brief introductory session for the students. A consent letter was sent home for parents or guardians detailing the research process and explaining that participation was optional. While all students received the REACH education program as part of the school curriculum, only students who obtained signed consent from their parent or guardian and provided assent were eligible to participate in the study. Paper-based questionnaires were administered by a research team member in person, both before and after the educational program. Students were asked to label their questionnaire with a unique code name that only they would know so that the pre-test and post-test could be matched while maintaining anonymity. School teachers were not involved in the research process and all questions about the research process were directed to the research team member. The post-test was administered within seven days of completion of the cannabis education module.

#### Data analysis

The questionnaires were scored out of 64 (i.e., there were 16 questions with 4 answers provided for each question for a total of 64 discrete answers), with one mark awarded for each correct answer. The data were analyzed descriptively and match-paired t-tests compared the difference between pre- and post-scores using IBM SPSS (version 26). Subgroup analyses examined the changes according to grade, gender, and school. Students who were absent on one of the test days and did not have a complete data set were removed from the analysis.

## Results

### Phase 1: Questionnaire development

Of thirty-four experts with knowledge about cannabis who were approached to participate, 24 completed the first round of the Delphi process (70.6%). The panel consisted of 6 health care professionals, 5 policymakers, 4 patients, 3 academics, and 6 students. Twenty-one experts continued participation through rounds 2 and 3 (87.5% retention) and 18 followed through to the last round (75% retention). The final round consisted of 6 health care professionals, 3 policymakers, 3 patients, 2 academics, and 4 students.

### Principal component analysis (PCA)

Seventeen codes were identified from the qualitative analysis and included as variables in the PCA, which identified three components. The first component consisted of four codes, entitled social consequences, substance misuse, familial support and cultural links which had a correlation of 0.985. These concepts revolved around effects of cannabis on the individual (including social exclusion and how to address cannabis use or overuse within a family), and the cultural importance of cannabis to some, and were collectively labelled ‘*impact of cannabis on the individual’*. The second component included 3 of the 17 codes (gaps in scientific evidence, different components of cannabis, and contraindications). This component was grouped as ‘*general information about cannabis’* as the themes involved scientific knowledge of cannabis and its side effects. The third component, which included concepts related to cannabis regulation (legislation) and harm reduction strategies, was termed ‘*cannabis harm reduction’*. The results of the PCA are depicted in Table 1.

**Table 1:** Principle component analysis.

Codes	Component 1: Impact of cannabis on the individual	Component 2: General information about cannabis	Component 3: Cannabis harm reduction
Background Information	-0.146	-0.247	0.192
Gaps in Scientific Evidence	-0.230	**0.842**	-0.126
Different Components	-0.192	**0.777**	-0.016
Different Forms or Strains	-0.139	0.180	0.304
Drug-drug interactions	-0.123	0.426	-0.076
Contraindications	-0.225	**0.624**	0.232
Physiological Consequences	-0.417	-0.256	0.335
Social Consequences	**0.958**	0.085	-0.189
Benefits	-0.139	-0.170	-0.400
Medical Use	-0.259	0.492	-0.229
Harm Reduction Techniques	0.613	0.161	**0.568**
Procurement	-0.073	-0.370	-0.357
Regulation	0.464	0.048	**0.378**
Substance Misuse	**0.958**	0.085	-0.189
Familial Support	**0.958**	0.085	-0.189
Cultural Links	**0.958**	0.085	-0.189
Reliable Resources	0.817	0.067	0.252

#### Questionnaire iterations

The questionnaire presented to the panel in round 2 consisted of 20 questions. Eight questions covered the impact of cannabis on the individual (component 1), 7 assessed general cannabis knowledge (component 2) and 5 questions were related to cannabis harm reduction (component 3). Each of the questions were analyzed to determine agreement amongst the participants and modifications were made accordingly. Sixteen questions in round 1 (80%) received agreement of 70% or greater, while four questions (20%) failed to meet the 70% threshold. These 4 questions were eliminated, with some parts of the questions incorporated into other questions of a similar theme. Some topics were felt to be highly important both in the agreement scores and open-ended feedback, including questions regarding the misconceptions of cannabis (Q1), terpenes and cannabinoids (Q2, Q6), cannabis potencies (Q4), harmful effects of cannabis (Q8, Q9, Q11), cannabis use below the age of 25 (such as Q12), and cannabis and travel (such as Q18). The questionnaire was shortened to 16 questions, and all received acceptable agreement scores in the subsequent rounds of the Delphi process. Agreement scores for Rounds 2 to 4 are presented in Table 2.

**Table 2:** Percent agreement based on percent of experts who agree and strongly agree with the inclusion of each round of questions on the CKAT.

Question	Percent Agreement (% strongly agree + agree)		
	Round 2	Round 3	Round 4
1	86.37	71.43	100
2	72.72	90.48	88.89
3	81.82	90.48	94.45
4	63.64	90.47	88.88
5	81.81	95.24	83.33
6	68.18	90.48	88.89
7	63.64	90.48	88.89
8	72.73	90.48	94.45
9	68.18	95.24	77.77
10	95.45	80.96	83.34
11	86.36	95.24	94.44
12	77.27	80.95	100
13	72.73	90.48	94.44
14	77.27	85.72	88.89
15	72.73	95.24	88.89
16	95.46	85.72	72.22
17	95.46		
18	90.91		
19	72.73		
20	81.82		

Refinements to the questionnaire were often suggested throughout the Delphi process to improve the degree of difficulty both with respect to the content and wording. For example, in round 2, phrases such as *onset of effect*, *harm reduction strategies*, and *adverse consequences* were criticized, and vague words such as *curative* (Q1) and *effective* (Q4) were modified. Subsequent suggestions included changing wording to improve inclusivity, consistency, and formatting. Efforts were made to keep the questionnaire generic so that it could be used across multiple settings without the need for continuous updating.

#### Final questionnaire

The final questionnaire consisted of 16 multiple true-false questions with a total of 64 items. Feedback was solicited from a few 7^th^ and 9^th^ grade students as to the format that the questionnaire should take. The students suggested that single true and false questions were too easy, and multiple-multiple choice (MMC) questions were recognized as too difficult. The students were indifferent to multiple choice (MC) and multiple true-false (MTF) questions. MTF allows for more items to be tested, with a smaller number of questions, hence it may help avoid participant fatigue and skipped questions [26, 27]. Also, MTF questions more accurately identify students with misunderstandings of concepts or incomplete understanding, as compared to multiple choice (MC) questions which only capture a student’s preferred answer and do not explore students’ thinking of the other answer options [26, 28]. Ultimately, the MTF format was chosen due to input from the students, the researchers’ experience, and because it allowed for knowledge of 64 separate items within only 16 questions to be tested in a period of less than 15 minutes. The questionnaire received a Flesch-Kincaid Grade Level score of 6.3, and a SMOG index score of 7.6. A copy of the questionnaire is attached as a S1 Appendix.

### Phase 2: Questionnaire testing

The pre-test was administered to 413 students in 7 schools in Saskatoon, SK. The study was interrupted by the Covid-19 pandemic and hence the post-test could not be completed in 3 schools. Therefore, the dataset consisted of 4 classrooms and 138 students. Six tests had unmatched codenames and could not be analyzed resulting in 132 total participants (96% response rate, 32% overall completion). Seventy-three 7^th^ grade students from Brunskill School and St. Lorenzo Ruiz Catholic School and 59 9^th^ grade students from Tommy Douglas Collegiate and Bishop James Mahoney High School participated, respectively. The demographics of the participants are shown in Table 3.

**Table 3:** Demographics of questionnaire respondents.

Breakdown according to school					
	Total *n* (%)	Tommy Douglas Collegiate *n* = 18 (13.6%)	Bishop James Mahoney High School *n* = 41 (31.1%)	Brunskill School *n* = 29 (22.0%)	St. Lorenzo Ruiz Catholic School *n* = 44 (33.3%)
**Gender**	Female *n* = 84 (63.6%)	18 (100%)	26 (63.4%)	14 (48.3%)	26 (59.1%)
	Male *n* = 46 (34.8%)	0	15 (36.6%)	14 (48.3%)	17 (38.6%)
	Other *n* = 2 (1.5%)	0	0	1 (3.4%)	1 (2.3%)
**Grade**	7 *n* = 73 (55.3%)	0	0	29 (100%)	44 (100%)
	9 *n* = 59 (44.7%)	18 (100%)	41 (100%)	0	0

Note: (%) in greyed area represent the percentage of the specific demographic within each school.

Table 4 summarizes the average pre-test, average post-test, and average post-pre test differences in Cannabis Knowledge Assessment Tool scores with the associated standard deviations (SDs). From Table 4, the overall average CKAT score increased from 46.2 (SD 5.5) to 50.7 (SD 4.6) indicating an average increase in CKAT score (mean post-pre test change 4.5; p<0.05), suggesting an average increase in student knowledge about cannabis after participating in the educational program. Subgroup analyses using match paired t-tests found statistically significant improvements within all subgroups as indicated in Table 4.

**Table 4:** Average pre-test, post-test, and post-pre test Cannabis Knowledge Assessment Tool (CKAT) scores with standard deviations (SDs) according to demographics.

Population	N	Mean Pre-test (SD)	Mean Post-test (SD)	Mean Post-Pre Change (SD)	P-value
**All students**	132	46.2 (5.5)	50.7 (4.6)	4.5 (4.3)	<0.05
**Grade 7**	73	44.1 (5.2)	49.3 (4.6)	5.2 (4.3)	<0.05
**Grade 9**	59	48.8 (4.7)	52.5 (3.9)	3.7 (4.3)	<0.05
**Female**	84	45.6 (5.5)	50.7 (4.5)	5.1 (4.6)	<0.05
**Male**	46	47.1 (5.3)	50.7 (4.8)	3.5 (3.7)	<0.05
**Tommy Douglas Collegiate**	18	47.4 (3.8)	51.0 (4.2)	3.6 (5.6)	n/a^a^
**Bishop James Mahoney High School**	41	49.4 (4.9)	53.2 (3.7)	3.8 (3.6)	<0.05
**Brunskill School**	28	45.2 (5.9)	49.5 (5.1)	4.2 (3.6)	<0.05
**St. Lorenzo Ruiz Catholic School**	44	43.3 (4.6)	49.1 (4.3)	5.8 (4.7)	<0.05

^a^Statistical analyses were not performed on sample sizes less than 25.

## Discussion

Changes to cannabis legislation have necessitated effective messaging around the safe use of cannabis. Survey tools have been developed to monitor trends in cannabis use [29] and capture perceptions of cannabis risk [15], but there is no mechanism for identifying gaps in public knowledge. Since this was the first attempt at developing a standardized tool to assess cannabis knowledge, a method that allowed us to gain expert opinion and reach consensus was deemed essential for increasing the content validity. The Delphi method allowed for a large and broad sample of experts to provide input into the questions, while upholding their anonymity to minimize bias and groupthink.

A total of 34 experts were originally invited to participate. For the 1^st^ round 24 experts participated providing open ended feedback, and this decreased to 21, 21, and 18 participants in subsequent rounds of feedback. The decrease as the rounds progressed is not unexpected, as the experts start to feel fatigue as more and more consensus starts to form [21]. The number of participants in the Delphi method can range from a minimum of 10 to a maximum of 50 to 100, and it is recommended to have the smallest number of participants possible that allows for a representative sample of different expert perspectives [30]. In our case, the experts who participated from start to finish included health care professionals, policymakers, patients, academics, and students, therefore wide representation was maintained.

For each round of questionnaire feedback, a threshold of 70% agreement was used to determine which questions required modifications or elimination, which is generally considered adequate [31]. Round 2 was the only time that some questions had a percent agreement lower than 70%, which was related to wording and difficulty of the questions. Round 3 had one question with much less agreement than other questions, and round 4 had most questions achieving greater than 80% agreement, and saturation had occurred with broad consensus on the questions being achieved. This is congruent with previous studies suggesting that normally the Delphi process takes 2 to 4 rounds [20, 21].

Since we aimed to create a generic tool that could be used across the public health spectrum, we needed to ensure that our tool was widely accessible. We included students on our expert panel and solicited their feedback on the questionnaire format. We also piloted the questionnaire with youth in the 7^th^ and 9^th^ grades, as data indicates that most youth either have heard about cannabis or have had exposure to it by this age [29, 32]. The student input ultimately helped shape the final questionnaire.

The questionnaire was piloted in four separate schools with 7^th^ grade and 9^th^ grade students, and results showed that knowledge improved in a statistically significant manner after a cannabis education program. This is encouraging, as with recent legalization for recreational use and increased interest in the medicinal use of cannabis, it is important that those who are at an impressionable age are knowledgeable about its effects. The improvements were consistent across each demographic with adequate sample size (grade, gender, school). The scores of the 9^th^ grade students were higher in the pre-test and the post-test as compared to the 7^th^ grade students. Since the REACH program has different modules for 7^th^ grade and 9^th^ grade students based on the Saskatchewan Ministry of Education’s health education curricular for each grade, the content variation of the program may explain this. Also, students in the 9^th^ grade may be exposed to more information about cannabis through media, peers, and their families as they were older when legalization occurred, and they have additional years of ‘real world experience’. No floor or ceiling effects were reported for the CKAT as no participants achieved a perfect or zero score. While the increase in knowledge scores were statistically significant, whether they were also educationally significant is unknown.

There are a few limitations to this study worth mentioning. This questionnaire was developed with input from a purposive sample; hence it may not be totally generalizable to the entire population. School teachers were not included on the expert panel, and they could have provided additional input into the appropriate wording and understandability for questionnaire development. This questionnaire was only piloted in 7^th^ grade and 9^th^ grade students in one urban centre; hence it is unknown how it might perform in other geographical centres across Canada, internationally, or in other age groups. While public and Catholic schools from different geographical locations in Saskatoon, Saskatchewan were studied, sampling bias may continue to exist. Only data from four of the seven schools were able to be gathered due to data collection occurring in March 2020 which was when COVID-19 initially resulted in the closure of in-person learning in Saskatoon, Saskatchewan. Finally, while measuring knowledge is an important aspect assessing the effectiveness of educational programs, we acknowledge that ‘knowing’ information does not necessarily translate to ‘doing’. Outcomes measures that capture behaviour change remain the holy grail for assessing public messaging and harm reduction campaigns.

## Conclusions

The Cannabis Knowledge Assessment Tool (CKAT) is the first known tool developed and tested to assess knowledge about cannabis. In a convenience sample of 7^th^ grade and 9^th^ grade students who participated in a cannabis educational program, knowledge about cannabis improved. Future studies are required to test usability and validity of the CKAT in other contexts.

## Supporting information

S1 Appendix CKAT questionnaire. (PDF)
