# Supplementary material for: The development of a Cannabis Knowledge Assessment Tool (CKAT)
Source: PLoS One. 2023 Sep 1;18(9):e0291113. doi: 10.1371/journal.pone.0291113 (PMC10473536; doi:10.1371/journal.pone.0291113)
Supplement: S1 Appendix — (PDF) [file pone.0291113.s001.pdf]

S1 Appendix. Cannabis Knowledge Assessment Tool (CKAT)

√ CHECK ONLY THE CORRECT ANSWERS (YOU MAY CHOOSE MORE THAN ONE ANSWER).

1. Which statements are **TRUE** regarding the compounds (ingredients) in cannabis? (check all of the correct answers)

- ☐ THC (tetrahydrocannabinol) is what causes people to “high”
- ☐ CBD (cannabidiol) is what causes people to feel “high”
- ☐ Terpenes are what cause people to feel “high”
- ☐ Cannabis used for medical conditions may contain both THC and CBD

2. Which statements are **TRUE** regarding cannabis terms? (check all of the correct answers)

- ☐ Cannabis is sometimes called “marijuana”, “weed”, or “pot”
- ☐ The terms “sativa” and “indica” refer to different cannabis plants
- ☐ Cannabis with a high amount of THC is called “hemp”
- ☐ The term “cannabinoids” refers to the compounds in cannabis, such as THC and CBD

3. Which statements about cannabis are **TRUE**? (check all of the correct answers)

- ☐ Cannabis is natural, so that means that it is safe to use
- ☐ Scientists and health care professionals know all of the potential harmful effects of cannabis
- ☐ Scientists and health care professionals know all of the potential benefits of cannabis
- ☐ More research is needed to know all the effects of cannabis

4. Which statements are **TRUE** regarding the effects of cannabis? (check all of the correct answers)

- ☐ Inhaling cannabis provides the fastest effects
- ☐ The effects one person feels may be different than the effects another person feels
- ☐ The time it takes to feel the effects of cannabis can be different for everyone
- ☐ People typically feel the effects of edible cannabis within 10 minutes

5. Based on what is known about potential harms, who should *avoid* using recreational cannabis? (check all of the correct answers)

- ☐ Someone who is pregnant or breastfeeding
- ☐ Someone that is older than 65 years old
- ☐ Someone who has a substance use disorder
- ☐ Someone with a personal or family history of psychosis (for example, schizophrenia)

6. Unless you are being treated for a medical condition by a health care professional, why is it important to *avoid* using cannabis before the age of 25? (check all of the correct answers)

- ☐ Using cannabis is not legal until you are 25
- ☐ Using cannabis before 25 may increase the risk of depression later in life
- ☐ You can become addicted to cannabis if you use it before 25, but not after
- ☐ The human brain does not fully develop until around the age of 25

7. People considering cannabis for a *medical condition* should: (check all of the correct answers)

- ☐ Speak to a health care professional (for example, doctors, pharmacists or nurses)
- ☐ Try cannabis from a licensed retailer
- ☐ Try some cannabis from their friends first to see if it may work
- ☐ Try edibles first

8. What are some of the potential *short-term* negative effects of using cannabis? (check all of the correct answers)

- ☐ Difficulty focusing and confusion
- ☐ Lung cancer
- ☐ Increased heart rate
- ☐ Slower reflexes

9. Which of the following ways of using cannabis may be harmful to someone's lungs? (check all of the correct answers)

- ☐ Smoking cannabis
- ☐ Eating cannabis
- ☐ Vaping cannabis
- ☐ Rubbing cannabis on their skin

10. If someone uses a large amount of cannabis for a long time and then suddenly stops, what are some of the withdrawal symptoms they might experience? (check all of the correct answers)

- ☐ Mood changes, such as increased anxiety
- ☐ Better ability to focus
- ☐ Difficulty sleeping
- ☐ There are no known withdrawal symptoms

11. Which statements are **TRUE** about recreational cannabis use in Canada? (check all of the correct answers)

- ☐ The legal age for using it in all provinces is 18 years old
- ☐ The laws for possession are the same in all Canadian provinces
- ☐ You can buy it from licensed online sellers
- ☐ You can grow as much as you want in your home if it is for personal use

12. Which statements are **TRUE** regarding the regulation of cannabis? (check all of the correct answers)

- ☐ You need medical authorization to purchase cannabis from a licensed retailer
- ☐ You can legally purchase recreational cannabis from anyone
- ☐ Licensed producers have similar rules for making both medical and recreational cannabis
- ☐ Stores that sell cannabis can only sell products that are guaranteed to have the same ingredients in them every time

13. Which statements are **TRUE** regarding Canadian cannabis laws? (check all of the correct answers)

- ☐ The maximum amount of cannabis you can carry in public is 30 grams of dried cannabis
- ☐ Drug-impaired driving is not allowed at any time
- ☐ You can use cannabis in a parked vehicle
- ☐ The laws for consuming cannabis in a public space are the same all across Canada

14. If cannabis is used, which decisions may be helpful for reducing harm? (check all of the correct answers)

- ☐ Avoid driving for at least six hours after using cannabis
- ☐ Buy cannabis from a licensed retailer
- ☐ Do not use cannabis until you are at least 25 years old
- ☐ Use cannabis with a lower THC content

15. Which statements are **TRUE** regarding cannabis and travel? (check all of the correct answers)

- ☐ Bringing cannabis into Canada is illegal
- ☐ Leaving Canada with cannabis is illegal
- ☐ You may not be able to enter the USA if you have used cannabis
- ☐ You can take cannabis to other countries if it is also legal there

16. Where should you seek out *reliable* information about cannabis? (check all of the correct answers)

- ☐ Health care professionals (for example, doctors, pharmacists or nurses)
- ☐ Government of Canada website
- ☐ Retail stores that sell cannabis
- ☐ Your peers who are using cannabis
